# Chloralamid as a Hypnotic

**Published:** 1898-03

**Authors:** 


### CHLORALAMID AS A HYPNOTIC.

Reynold W. Wilcox, M.D., LL.D., Professor of Medicine and Therapeutics, says in *The Post-Graduate*, November, 1897:

"Chloral is the most popular hypnotic, but it is one which most frequently gives rise to habit.

"There is a safe derivative of chloral, however, chloralamid. The introduction of the amide radical neutralizes to a considerable extent the depressing action on the heart. It is fairly insoluble, and is therefore more prolonged in its action. It is far safer than chloral. It is difficult to form a habit with chloralamid, yet I know of one instance in which the patient developed the habit after taking it without my knowledge for a year. The habit was cured without great difficulty.

"Probably the most potent hypnotic is paraldehyde; next comes chloralamid; then pellotin, and lastly trional. Sleep follows most quickly after pellotin, next after paraldehyde, then after chloralamid, and lastly after trional. With moderate doses the longest sleep is obtained from trional; next comes paraldehyde; then pellotin, and lastly chloralamid. The danger of a habit from pellotin is extremely slight; it is a little greater from chloralamid, and there is very great danger from paraldehyde, as shown by the published reports. Chloralamid seems to be the safest of them all; next comes pellotin; next paraldehyde, and the most dangerous for continuous administration is trional."—*Med. Review of Reviews.*

